# Moderating Effect of Family Support on the Mediated Relation Between Negative Life Events and Antisocial Behavior Tendencies *via* Self-Esteem Among Chinese Adolescents

**DOI:** 10.3389/fpsyg.2020.01769

**Published:** 2020-07-24

**Authors:** Feifei Gao, Yuan Yao, Chengwen Yao, Yan Xiong, Honglin Ma, Hongbo Liu

**Affiliations:** ^1^Department of Health Statistics, School of Public Health, Jinzhou Medical University, Jinzhou, China; ^2^Medical College, Shandong University, Jinan, China; ^3^Middle School of Ying-Li Town, Heze, China; ^4^Hospital of Xi-He Town, Chengdu, China; ^5^Department of Health Statistics, School of Public Health, China Medical University, Shenyang, China

**Keywords:** adolescent, antisocial behavior, self-esteem, family support, negative life events

## Abstract

Adolescents are particularly prone to antisocial behavior. The promoting effect of negative life events on antisocial behavior has been well-documented. However, the internal influence mechanisms between negative life events and antisocial behavior tendencies in adolescents are still unknown. The aim of this study was to explore the mediation effect of self-esteem and the moderated mediation effect of family support between negative life events and antisocial behavior tendencies in 8,958 adolescents who were selected from three Chinese provinces. Robust maximum likelihood estimator (MLR) of a structural equation model (SEM) was applied to test the mediation model and the moderated mediation model. The results revealed that negative life events had a positive effect on antisocial behavior tendencies in adolescents, with a direct effect of 0.082 (95% CI: 0.052, 0.111) and an indirect effect *via* self-esteem of 0.168 (95% CI: 0.146, 0.191). Negative life events had a 67.20% effect on antisocial behavior tendencies, where self-esteem showed mediation. The indirect effect was 2.049-fold greater than the direct effect. Furthermore, the effect of latent interaction of subjective family support and negative life events on self-esteem was negatively significant (*β* = −0.018, *p* = 0.032, 95% CI: −0.035, −0.002). The indirect effect of negative life events was greater, where subjective family support was below 1 SD of the mean (conditional indirect effect = 0.227, 95% CI = 0.200, 0.255) than where it was above 1 SD of the mean (conditional indirect effect = 0.203, 95% CI = 0.177, 0.229). The moderated mediation effect index was −0.012, *p* = 0.033. Moderated mediation showed that the mediated path was less evident in the students who had greater subjective support from family. The results of the current study demonstrated the important role that self-esteem and subjective family support played in minimizing the adverse effect of negative life events on antisocial behavior development of adolescents. These findings have important implications for preventing antisocial behavior in adolescents by developing interventions aimed at enhancing their self-esteem and providing support-skill training to parents aimed at improving subjective family support of adolescents.

## Introduction

Antisocial behavior is mostly regarded as consisting of attitudes that go against the grain in society and/or disregard the rights of others (Molero Jurado et al., 2017). Antisocial behavior can be harmful to others or their homes and possessions. Adolescents, their family and society can be burdened with substantial costs in regard to physical harm and emotional duress, with financial hardship as well (Cook et al., 2015; Sawyer et al., 2015). Antisocial behavior has become a critical concern for public health worldwide, and it is especially prevalent in youths. Antisocial behavior tendencies are seen primarily in adolescence with a high incidence (Armelius and Andreassen, 2007), so this stage in life has become very interesting for the study of this deviant behavior. A tremendous portion of adolescents display some type of antisocial behavior (Tremblay, 2010). Children who show antisocial behavior tendencies are prone to a number of detrimental issues in their development, including dropping out of school, criminal activity, psychological problems, and substance abuse (Tieskens et al., 2018; Sharman et al., 2019). Antisocial behavior tendencies in the young are known to have negative consequences, so determining the factors associated with the development of antisocial behavior in young people is still a hot research topic. There is a need to elucidate the risk factors and related mechanisms of antisocial behavior tendencies in adolescents to develop prevention as well as intervention measures.

A positive life environment is a protective factor against developing a problem behavior and its persistence as well. On the other hand, negative environmental factors can induce an antisocial behavior, where they are considered to be strong predictors of such behavior in youths (Sousa et al., 2011; Murray et al., 2012). Many studies indicate that adverse events in youth’s formative years have long-term physical and mental health consequences (Norman et al., 2012; Gilbert et al., 2015). Exposure to adversity during development leads to, among other issues, a substantial increase in the probability of developing antisocial behavior (Mackey et al., 2017). Therefore, researchers are looking closely at how to mitigate the detrimental effects of negative life events on antisocial behavior tendencies in these adolescents. Antisocial behavior tendencies in adolescence, and its continuation in later life, are believed to involve an interaction of psychological vulnerability and environmental factors (Álvarez-García et al., 2019). Regarding psychological resources, self-esteem is thought to be an important internal resource, which has a significant effect in promoting children’s development (Gao et al., 2019). Self-esteem is usually defined as an emotional evaluation of the individual himself/herself (Park et al., 2019). Individuals who have high self-esteem usually have enough resources to cope with daily stresses. They are more confident in life and will face difficulties bravely, which makes them less susceptible to emotional depletion and personality problems. Earlier studies showed that self-esteem is negatively associated with antisocial behavior (Barry et al., 2003; Jia et al., 2020), indicating that high self-esteem is less likely to lead to antisocial behavior tendencies. Besides, previous studies have confirmed the mediating role of self-esteem between negative life events and negative emotions (Han et al., 2018; Sarubin et al., 2020), as well as a mediating role between perceived parenting and antisocial behavior tendencies (Hunter et al., 2015). Both the correlation of negative life events with self-esteem and the correlation of self-esteem with antisocial behavior tendencies have been studied. However, the comprehensive relationship of negative life events, self-esteem, and antisocial behavior tendencies has not yet been studied, and the mediating role of self-esteem between negative life events and antisocial behavior tendencies has not yet been confirmed. We may not be able to change the negative life events that the individual has experienced in a short time. However, if there is evidence demonstrating the mediation effect of self-esteem between negative life events and antisocial behavior tendencies, increasing self-esteem among adolescents could prevent the development of antisocial behavior, since the promoting effect of negative life events on antisocial behavior tendencies could be offset to some extent. Importantly, self-esteem is not a static phenomenon; rather, it is a dynamic process that can be altered at any time. Previous studies have confirmed that self-esteem could be improved through interventions (Nosek et al., 2016; Zangirolami et al., 2018). Accordingly, it will be of important theoretical and practical value to lessen the harmful effect of negative life events on antisocial behavior tendencies in adolescents, once the mediating role of self-esteem in the relationship between negative life events and antisocial behavior tendencies is established.

The effect of negative life events on adolescent antisocial behavior tendencies maybe mediated by self-esteem, and adolescents are sensitive to adverse events in different ways. According to the social control theory, an individual’s connection with society is critical in determining their activities (Wang et al., 2017). Adolescents’ relationship with family is important in preventing them from getting involved with delinquent activity. Prior research indicates that family support has positive effects on children (Schofield et al., 2012), which is inversely associated with antisocial behavior developing in youths (Piquero et al., 2009; Cutrín et al., 2017). Previous studies have made valuable contributions. However, there are few studies with large samples that take a look at how family support influences the effect of adversity events on antisocial behavior tendencies in adolescents. Previous research has indicated that there is evidence that family support may reduce stress among youths (Wills et al., 1992; Chukwuorji et al., 2017), but little is known about how family support may act as a protective factor against antisocial behavior tendencies. Based on a functional model of social support processes (Wills et al., 1992), family support allows adolescents to cope more with the usual challenges in life, especially subjective family support of adolescents. Subjective family support may be critical in improving adolescents’ ability to deal with various life stressors (Chukwuorji et al., 2017; Manczak et al., 2018), which may lessen the adverse effect of negative life events on self-esteem and social behavior. In current research, we predicted that subjective family support can have a stress-moderating effect in relation to self-esteem and then deter antisocial behavior tendencies from developing. The specific prediction is an interaction of negative life events and family support, such that the effect of negative life events on self-esteem is mitigated or abolished for adolescents with greater subjective family support. Since self-esteem may mediate the effect of negative life events on antisocial behavior tendencies, this study tests a hypothesis that the indirect association between negative life events and antisocial behavior tendencies *via* self-esteem will be moderated by family support.

**Figure 1:**
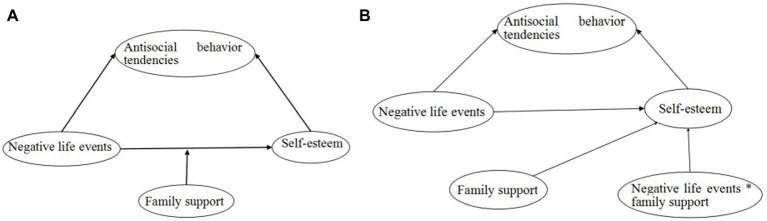
The theoretical hypothesis model of the present study (**A**: model diagram; **B**: statistical diagram).

According to the stress-support model and our literature review, antisocial behavior tendencies depend on the interaction of opposing factors that promote vulnerability or protection. In our study, the main factor predisposing susceptibility was negative life events, which can pose an increased risk to developing antisocial behavior in adolescents. The protective factors were family support and self-esteem, which could have an increased favorable influence, resulting in a lower risk of antisocial behavior development. The stress-support model specifically predicts that antisocial behavior tendencies are based on the interplay of factors that promote vulnerability or protection. The aim of our study was: (a) to see if self-esteem influences how negative life events affect adolescent antisocial behavior tendencies and (b) to determine if self-esteem mediation of the indirect association between negative life events and antisocial behavior tendencies is moderated by family support. A moderated mediation model based on these two research questions was thus investigated (Figure 1). Therefore, we proposed the hypotheses below:

Hypothesis 1: Self-esteem will mediate the link between negative life events and antisocial behavior tendencies in adolescents.
Hypothesis 2: Family support will moderate the mediation effect of self-esteem between negative life events and adolescent antisocial behavior tendencies.

## Materials and Methods

### Participants and Procedures

We selected 8,958 adolescents (6,002 middle school students and 2,956 high school students) from Sichuan, Henan, and Shandong provinces in China. First, we randomly selected one province from Eastern China (Shandong province), Central China (Henan province), and Western China (Sichuan province). Next, we randomly selected one city (Nanchong in Sichuan, Zhoukou in Henan, and Heze in Shandong) from each province. Finally, we choose two rural middle schools and one high school randomly in each city sampled. All students in the selected schools were participants of this cross-sectional study. Each student attended a 30-min session to complete the measures in their respective classrooms alone in the absence of teachers. This study was carried out in accordance with the Declaration of Helsinki. Approval to conduct the study was granted by the research ethics committee at Jinzhou Medical University [No: 2016-08]. Participation was voluntary. Voluntary informed consent was obtained from the participants, their parents, or legal guardians, and the principals of the sampled schools before the administration of any study-related questionnaires. We clearly stated that all their responses were collected anonymously and kept confidential, only to be used for research. They were allowed to drop out from the study whenever.

A total of 9,675 students were enrolled in this investigation, with 8,958 returning a complete and qualified questionnaire. Finally, 8,958 students were qualified adolescents in the present study, with 2,661 being from Sichuan, 4,353 from Henan, and 1,944 from Shandong.

### Measures

#### Negative Life Events

Negative life events were determined with the Adolescent Self-Rating Life Events Check List (ASLEC) (Xin and Yao, 2015). The scale evaluates the stressful life events that an individual has lived through in the last 6 months, in terms of frequency and intensity. This is a six-point Likert-type scale comprising 27 items concerning five dimensions, including interpersonal relationship, study pressure, punishment, bereavement, and change for adaptation. An example item of interpersonal relationship stressful events was “Dispute with a classmate or close friend.” An example item of study pressure events was “Failed or did poorly in the exam.” An example item of punishment events was “Beaten and abused by parents or others.” An example item of bereavement events was “The death of a close relative or friend.” An example item of adaptation stressful events was “The daily routine (diet, rest, etc.) changed significantly.” The questions are answered according to the following scale: 1 (did not occur), 2 (no effect), 3 (mild effect), 4 (moderate effect), 5 (severe effect), and 6 (very severe effect), where the scores were 0 (no occurrence), 0 (had no effect), 1 (mild effect), 2 (moderate effect), and 3 (severe effect) to 4 (very severe effect). A higher score indicated more stressful negative life event. A confirmatory factor analysis (CFA) was performed to confirm its factor structure. Since the scale comprised too many original items, item parceling was conducted, which was a recommended method to deal with the long scale (Yang et al., 2010). Thus, negative life events comprised five dimensions as their five indicators. The CFA model demonstrated a satisfactory fit [*χ*^2^/*df* = 20.487, *p* < 0.001, CFI = 0.947, TLI = 0.942, SRMR = 0.061, RMSEA = 0.059 (90% CI: 0.046, 0.068)], showing that this scale was suitable for the sample studied. The composite reliability of the assessment tool (0.7903), along with Cronbach’s alpha (0.918) and average variance extracted (0.5631), suggested that the internal quality of this model was satisfactory.

#### Self-Esteem

Five items were selected from the Rosenberg self-esteem scale to assess self-esteem in the present sample (Rosenberg, 1965). An example item was “I think I have a lot of strengths.” There are four possible answers for each item indicating the degree of agreement, where 1 indicates strongly disagree to 4 indicating strongly agree, and this scale has been validated in adolescents to assess their self-confidence and self-satisfaction (van Dijk et al., 2011; Romera et al., 2016). The final score for the five items determines the self-esteem level, where a higher level of self-esteem is indicated by a higher score. This scale showed CFA fit indices indicating that the fit was acceptable [*χ*^2^/*df* = 27.886, *p* < 0.0001, CFI = 0.965, TLI = 0.926, SRMR = 0.055, and RMSEA = 0.046 (90% CI: 0.037, 0.058)]. Cronbach’s alpha (0.905), composite reliability (0.7346), and average variance extracted (0.4861) were also acceptable, demonstrating that the internal quality of the model was suitable.

#### Family Support

The family support scale was selected from the Resilience Scale for Chinese Adolescent (RSCA) (Hu and Gan, 2008), which was one of the dimension of RSCA to assess one’s perception of family support by examining how he or she regards the supports from their parents and other family members. It comprises six Likert-type items. Examples of item were “My parents always encourage me to do my best,” “My parents respect my opinion very much,” etc. A five-point Likert-type scale is used to score each item, where 1 indicates completely unmatched and 5 indicates completely matching, according to the frequency of feeling satisfied with each item. High scores of the scale represent high levels of family support. The reliability and validity of this scale were acceptable in our sample according to Cronbach’s alpha (0.931), composite reliability (0.679), and average variance extracted (0.452). Additionally, the CFA model was a good fit model, where the fit indices of the scale were as follows: *χ*^2^/*df* = 8.249, *p* < 0.0001, CFI = 0.964, TLI = 0.959, SRMR = 0.049, and RMSEA = 0.043 (90% CI: 0.036, 0.054).

#### Antisocial Behavior Tendencies

Antisocial behavior tendencies were measured using six Likert-type items, which were components of the social adjustment scale for adolescents (Zhou and Zhang, 2008). The scale evaluates a sort of deviate behaviors that the participant has experienced, such as fighting, violating school rules, ignoring social norms, etc. An example of the item was “I often fight with others.” Scores go from 1 (strongly disagree) to 5 (strongly agree) Likert scale, according to the degree of agreement with which the respondent has experienced. A higher score indicates a higher level of antisocial behavior tendencies. The fit indices of the CFA model [*χ*^2^*/df* = 8.262, *p* < 0.0001, CFI = 0.968, TLI = 0.943, SRMR = 0.064, and RMSEA = 0.053 (90% CI: 0.042, 0.060)] suggested that the scale was acceptable in the current study. On the basis of composite reliability (0.751) and the average variance extracted (0.394), together with Cronbach’s alpha (0.932), we concluded that the sub-scale had an acceptable reliability and validity in our sample.

### Statistical Analysis

SPSS 21.0 software package and Mplus version 7.4 were used to analyze the data. The multiple imputation method was used to deal with missing data, while the percentage of missing data of 8,958 qualified questionnaires was 7.23%. In the preliminary analysis phase, the reliability and validity of the scales were determined using Cronbach’s alpha, composite reliability, and average variance extracted after CFA, which was conducted in Mplus 7.4. Cronbach’s alpha was greater than 0.90, indicating good reliability (Li et al., 2019). Composite reliability was greater than 0.60, and average variance extracted was greater than 0.50, indicating that each measurement construct had great convergence validity, where an acceptable average variance extracted can be between 0.36 and 0.50 (Fornell and Larcker, 1981). The overall fit of CFA was assessed using *χ*^2^/*df*, the comparative fit index (CFI), Tucker-Lewis index (TLI), the standardized root mean square residual (SRMR), the root mean square error of approximation (RMSEA), and the 90% confidence interval (CI) of RMSEA. SRMR and RMSEA ranging from 0 to 1 with a 0.08 cut-off for adequacy and a 0.05 cut-off being a more stringent value for goodness-of-fit, while 0.90 or greater suggesting a good fit for all the other indices (Tong and Bentler, 2013; Monroe and Cai, 2015).

On the basis of the theoretical hypothesis model (Figure 1), a structural equation model (SEM) was used to determine the moderated mediation association between negative life events, family support, self-esteem, and antisocial behavior tendencies. Considering the categorical nature and the non-normal distribution of the descriptive sat for the questionnaire items, robust maximum likelihood estimator (MLR) was performed to determine SEM in Mplus 7.4, which could provide values for the observed variables with SEs showing robustness to non-normality (Muthén and Muthén, 2010). The same fit indices as CFA models were used to evaluate the goodness-of-fit of SEM. The hypothesized model was tested in two steps. First, the mediation model without the hypothesized interaction was assessed. The mediation of self-esteem was indicated if the indirect effect of negative life events on antisocial behavior tendencies was significant. The effect size of mediation model was measured using the traditional effect size measures (*R_M_* and *P_M_*) together with the standardized indirect effect, where *R_M_* is the indirect effect to direct effect ratio and *P_M_* is the indirect effect to the total effect ratio (Preacher and Kelley, 2011). Next, the moderated mediation model with latent interaction was tested; while the latent interaction of family support and negative life events was created using latent moderated structural equations (LMS) in Mplus 7.4, after standardized family support and negative life events. A significant moderated mediation effect of family support could be established if the 95% CI of the interaction did not contain 0. To properly interpret it, the conditional indirect effect of negative life events on antisocial behavior tendencies *via* self-esteem was plotted for low, medium, and high family support values (high family support: values above mean + 1 SD; medium family support: values of mean ± SD; low family support: values below mean − 1 SD) (Hayes and Rockwood, 2017). The index of moderated mediation effect was used to measure the effect size of the moderated mediation model. A two-tailed *p* < 0.05 was considered statistically significant. In addition, age, gender, study site (Sichuan/Henan/Shandong), school type (middle school/high school), whether or not live with parents (both parents/mother only/father only/neither), and education level of parents were controlled for all SEM models, while we analyzed the mediation effect of self-esteem and the moderated mediation effect of family support.

## Results

### The Basic Characteristics of Subjects

The present study included 8,958 adolescents, of which 49.18% were males and 50.82% were females. The participants were from Henan province (48.59%), Sichuan province (29.71%), and Shandong province (21.70%). They were 10–17 years old [mean = 15.53 ± 2.23 (SD)]. A third of the participants (33.00%) were selected from high schools and 67.00% were from middle schools. The education level of the parents of participants was generally very low, where 54.66% of the fathers just had a middle school education and over half (53.18%) of the mothers only a primary school education or less. Around 47.35% of the adolescent lived with both parents in the past 6 months and 36.88% lived with neither of parents, while 13.69% lived with mother only and 2.08% with father only.

### Correlation Analysis Between Negative Life Events, Family Support, Self-Esteem, and Antisocial Behavior Tendencies

Pearson correlations and partial correlations were performed to analyze the correlation between two studied variables, while partial correlations were conducted after controlling for demographic characteristics (age, gender, study site, school type, whether or not live with parents, and education level of parents), and other studied variables. The results are tabulated in Table 1. Antisocial behavior tendencies of adolescents were positively related to negative life events, while inversely related to self-esteem and family support. The relationship between self-esteem and family support was also positive, while the variable negative life events were significantly and inversely related to self-esteem and family support.

**Table 1:** Correlation analysis between negative life events, self-esteem, family support, and antisocial behavior tendencies.

Variable	1 *Pearson r* (*Partial r*)	2 *Pearson r* (*Partial r*)	3 *Pearson r* (*Partial r*)	4 *Pearson r* (*Partial r*)
Negative life events (1)	1.000			
Family support (2)	−0.312(−0.348)^**^	1.000		
Self-esteem (3)	−0.201(−0.198)^**^	0.291(0.321)^**^	1.000	
Antisocial behavior tendencies (4)	0.200(0.213)^**^	−0.245(−0.250)^**^	−0.252(−0.282)^**^	1.000

** *p* < 0.001.

### Mediation Effect of Self-Esteem

**Figure 2:**
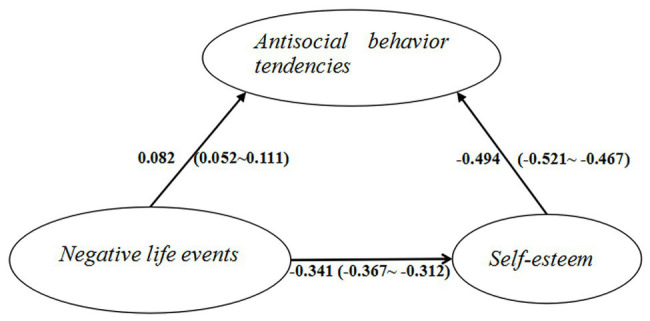
The mediation model of negative life events, self-esteem, and antisocial behavior tendencies. (The standardized direct effects and 95% CI between variables are presented here. Paths of the control variables are omitted for clarity).

To test the mediation effect of self-esteem between negative life events and antisocial behavior tendencies, SEM was used with self-esteem as a mediator of the effects of negative life events on antisocial behavior tendencies (Figure 2). The model fit was good [*χ*^2^*/df* = 9.48, *p* < 0.001, CFI = 0.944, TLI = 0.923, SRMR = 0.051, and RMSEA = 0.047 (90% CI: 0.034, 0.061)]. This model suggested that negative life events had a negative effect on self-esteem (*β* = −0.341, *p* < 0.001, 95% CI: −0.367, −0.312). Self-esteem was negatively related to antisocial behavior tendencies (*β* = −0.494, *p* < 0.001, 95% CI: −0.521, −0.467), while experiencing negative life events was positively related to antisocial behavior tendencies (*β* = 0.082, *p* < 0.001, 95% CI: 0.052, 0.111). The indirect effect of negative life events on antisocial behavior tendencies *via* self-esteem was 0.168 (*p* < 0.001, 95% CI: 0.146, 0.191). There were two paths by which negative life events affected antisocial behavior tendencies. First, negative life events affected antisocial behavior tendencies directly, with the direct effect (0.082) accounting for 32.80% of the total effect (0.250). Second, negative life events affected antisocial behavior tendencies indirectly through self-esteem, while the indirect effect (0.168) accounted for 67.20% of the total effect. The indirect effect was 2.049-fold greater than the direct effect (Table 2). These results demonstrated the mediating role of self-esteem between negative life events and antisocial behavior tendencies.

**Table 2:** The mediation effect size of self-esteem in the relationship between negative life events and antisocial behavior tendencies.

Path	Standardized effect	*p*	*P_M_* (%)	*R_M_*
Negative life events → Antisocial behavior tendencies	0.082	<0.001	32.80	---
Negative life events → Self-esteem → Antisocial behavior tendencies	0.168	<0.001	67.20	2.049
Total	0.250	<0.001	100.00	2.049

### Moderated Mediation Effect of Family Support

**Figure 3:**
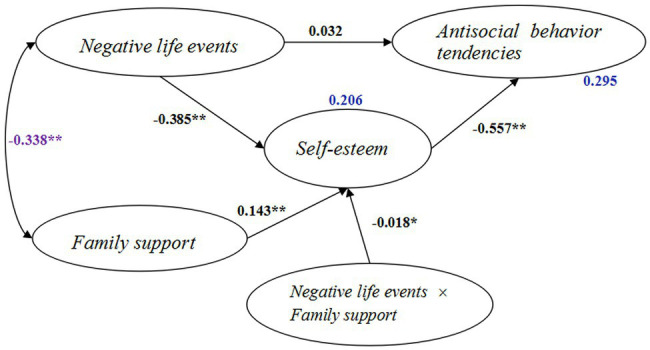
The moderated mediation model. (Standardized coefficients are presented here. Paths of the control variables are omitted for clarity. ^*^p < 0.05, ^**^p < 0.01).

**Figure 4:**
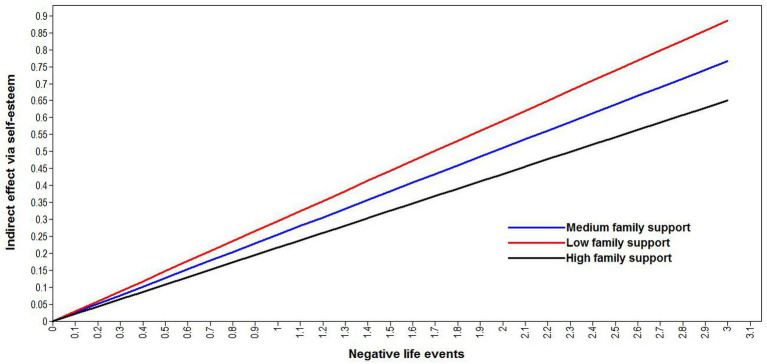
The conditional indirect effect of negative life events on antisocial behavior tendencies *via* self-esteem for each combination of low, medium, and high family support values. (High family support: family support values above mean + 1 SD; medium family support: family support values of mean ± 1 SD; low family support: family support values below mean − 1 SD).

We tested the moderated mediation model with the latent interaction of family support and negative life events (Figure 3). The results revealed that the direct link between negative life events and antisocial behavior tendencies was no longer significant (*β* = 0.032, *p* = 0.091, 95% CI: −0.005, 0.069). However, other paths that included the interaction term showed significance, as seen in Figure 3. Negative life events still had a negative effect on self-esteem (*β* = −0.385, *p* < 0.001), while self-esteem was negatively related to antisocial behavior tendencies (*β* = −0.557, *p* < 0.001). Negative life events had an indirect effect on antisocial behavior tendencies through self-esteem that was still significant (indirect effect = 0.214, *p* < 0.001, 95% CI = 0.191, 0.239), thus still supporting the notion that self-esteem mediated the effect of negative life events on antisocial behavior tendencies. Moreover, the effect of latent interaction of family support and negative life events on self-esteem was negatively significant (*β* = −0.018, *p* = 0.032, 95% CI: −0.035, −0.002), suggesting the negative moderating role of family support on the effect of negative life events on self-esteem. Therefore, family support moderated the mediation effect of self-esteem on the relationship between negative life events and antisocial behavior tendencies. The moderated mediation model explained 29.5% of the variance of antisocial behavior tendencies. We then plotted the conditional indirect effect of negative life events against antisocial behavior tendencies for each combination of low (below mean − 1 SD), medium (mean ± SD), and high (above mean + 1 SD) family support values. As seen in Figure 4, the indirect relationship between negative life events and antisocial behavior tendencies *via* self-esteem appeared more evident when family support was low than when it was high. The conditional indirect effect of negative life events on antisocial behavior tendencies through self-esteem for different family support level is tabulated in Table 3. A stronger indirect effect of negative life events was seen with low family support (conditional indirect effect = 0.227, 95% CI = 0.200, 0.255) than with high family support (conditional indirect effect = 0.203, 95% CI = 0.177, 0.229). The index of moderated mediation effect was −0.012, *p* = 0.033. These results verified the moderated mediation effect of family support on the relationship between negative life events and antisocial behavior tendencies *via* self-esteem.

**Table 3:** The conditional indirect effect of negative life events on antisocial behavior tendencies through self-esteem for different family support levels.

Family support level	Indirect effect	Standard error	*p*	95% CI
High family support*^a^*	0.203	0.013	<0.001	(0.177, 0.229)
Medium family support*^b^*	0.215	0.013	<0.001	(0.191, 0.240)
Low family support*^c^*	0.227	0.014	<0.001	(0.200, 0.255)
Index of moderated mediation effect	−0.012	0.005	0.033	(−0.023, −0.003)

a Family support values above mean + 1 SD.

b Family support values of mean ± 1 SD.

c Family support values below mean − 1 SD.

## Discussion

The effect of negative life events on antisocial behavior has gained empirical support. But the mechanisms underlying related mediating and moderating effects are still unclear. This study put forth a moderated mediation model in a sizable sample of adolescents to test whether self-esteem mediates the effect of negative life events on antisocial behavior tendencies, and whether family support moderates the indirect effect of negative life events on antisocial behavior tendencies *via* self-esteem. The results obtained in the current study supported our hypothesis.

Consistent with previous research (Mackey et al., 2017), this study found that negative life events positively correlated with antisocial behavior tendencies in adolescents, which again confirmed the adverse effect of negative life events on adolescent development. However, it is of little practical implication to prevent antisocial behavior by reducing negative life events, as we may not be able to change the negative life events that an individual has experienced. On the contrary, it will be of important practical value to lessen the harmful effect of negative life events on antisocial behavior tendencies in adolescents. Despite the well-recognized connection between antisocial behavior and negative life events (Norman et al., 2012; Gilbert et al., 2015; Sarubin et al., 2020), many individuals who deal with highly stressful situations do not develop antisocial behavior, so the particular characteristics of this association is still uncertain. As a result, there is an urgent need to clarify the influence factor and internal mechanism of negative life events leading to antisocial behavior. However, there is little evidence indicating the underlying influencing mechanisms in the association of negative life events with antisocial behavior tendencies. This is the first study to examine the mediator and moderator between negative life events and antisocial behavior tendencies in adolescents, thus extending and enriching the research on antisocial behavior. The stress-support model sees antisocial behavior as being associated with an interplay of protective and vulnerability factors. The present study treated negative life events as the primary vulnerability factor that puts the youth at a higher risk of developing antisocial behavior. The protective factor is self-esteem, which may decrease risk for antisocial behavior (Costello et al., 2018). In line with our first hypothesis, our results here showed that negative life events correlated inversely with adolescent self-esteem, which itself was negatively associated with antisocial behavior tendencies in adolescents; that is, self-esteem intervened in the effect of negative life events on antisocial behavior tendencies in adolescents. Therefore, adolescents showing more self-esteem are less prone to developing antisocial behavior. These results are consistent with previous research in problem behavior, demonstrating that those with more self-esteem will probably cope better with stressful events, resulting in fewer psycho-social problems (Barry et al., 2003; Jia et al., 2020). Consequently, enhanced self-esteem may be one of the mechanisms that explain why some adolescents who experience many negative life events are still less prone to antisocial behavior. The current study showed that most of the adverse effect of negative life events on antisocial behavior tendencies is mediated by self-esteem. This result is consistent with the idea that self-esteem can be viewed as an important psychological resource and motivating factor for preventing antisocial behavior (Gao et al., 2019). Although it is impossible to change the negative life events that an individual has experienced, antisocial behavior tendencies can be avoided once self-esteem is improved, due to the negative effect of self-esteem on antisocial behavior tendencies. In addition, previous studies have demonstrated that self-esteem can be managed and developed (Nosek et al., 2016; Zangirolami et al., 2018). Therefore, considering the buffering action of self-esteem on the impact of negative life events, it will be of great practical significance to prevent the development of problem behavior of adolescents, if managers (for example, teachers, government workers, etc.) could focus on promoting self-esteem and training among adolescents. The results of the present study contribute to the potential use of psychological tools in dealing with the adverse effect of negative life events on antisocial behavior tendencies. These results highlight the importance of self-esteem in combating the consequences of negative life events, helping individuals to stay healthy.

In line with our second hypothesis, our research showed that family support moderated the indirect effect of negative life events on antisocial behavior tendencies through self-esteem. Prior research has stressed that family support may be essential in preventing potential emotional disorder and psychological problems of children and young adolescents facing adversity (Oliva et al., 2009). The current study found that subjective family support weakened the adverse effect of negative life events on self-esteem and subsequent antisocial behavior tendencies as well. This finding is consistent with the stress-buffering model, which argues that adolescents with strong family support, who experience many stressful life events, would not show psychological maladjustment (Jose and Kilburg, 2007). For example, those who have greater subjective family support rely more on approach-coping, and less on avoidance-coping (Romero et al., 2015). Family support could enhance their belief in resources for coping with potentially stressful situations to lower their emotional impact or to prevent emotional disruptions from becoming a problem behavior (Schofield et al., 2012). Therefore, adolescents with strong subjective family support will make appraisals of life events in a less negative manner, and the adversity effect of life events on self-esteem will be weakened. The present study demonstrated the stress-moderating effect of family support in relation to self-esteem and antisocial behavior tendencies of adolescents. Family support decreased the indirect effect of negative life events on antisocial behavior tendencies in adolescents, protecting them from the possibly detrimental effect of stressing events, which points to the need to enhance subjective family support during adolescence. Consequently, family support can be viewed as a way to protect youths from the harmful effects of negative life events on adolescent psychological and social behavior adjustment. Thus, our study expands our understanding of antisocial behavior management by elucidating the boundary condition where negative life events and low self-esteem can effectively contribute to antisocial behavior tendencies. It is probable that some parents relax their involvement and support in their children at adolescence, since they consider that they no longer play an important role (Oliva et al., 2009). Contrarily, our study points out the critical role that family support appears to have in the behavioral adjustment of youths, which protects them from some negative effects of stressing events in life. Accordingly, parents should be made aware of their influence on the healthy development of adolescents, and should be guided in improving their parenting skills by enhancing their communication, affection, and monitoring. This support can be more effective through the use of programs for communication training (Wills et al., 1992), which could be adapted to prevention programs by personal training or other ways, such as online training. Adolescents with a supportive family could show more resistance to the negative effects of some adverse situations that may often occur in adolescence.

This study extends our knowledge of how self-esteem and family support play a complex role in the effect of negative life events on antisocial behavior tendencies in adolescents. This study points out important implications for preventing antisocial behavior in adolescents. The results show us the importance of self-esteem and subjective family support to the development of adolescent, even though some faced with a variety of stressful life events. To minimize the adverse effect of life events, there is an urgent need to develop interventions aimed at enhancing self-esteem and pay enough attention to the important role of subjective family support in the development of psychological and social function among adolescents. This study clearly provides interesting results and makes important contributions to the field, but it had several limitations. First, the causality of the variables was difficult to prove using the present cross-sectional study. Longitudinal designs may be considered in any expansion of the present work to better understand the relationship of the variables. Second, the information bias may be unavoidable, since all the information was obtained through self-reporting by the adolescents. It is probable that such self-reported information from adolescents was biased by social desirability. Further studies should work with more data sources, such as parents, teachers, and peers. Besides, each studied constructs of the present research was measured by one single scale. Multiple different scales should be used to measure the same structure in future studies, which can complement each other to give a better picture of the studied constructs. Furthermore, this study only measured certain demographic as potential confounding variables. Additional confounding variables, such as socioeconomic factors, should be included in future studies. And, the inter-individual patterns of the obtained models would be worth to explore in future studies, so as to enrich the practical implications of our research. In addition, it is possible that other social supports (e.g., close friends or teachers) would buffer the lack of subjective family support. Thus, a very interesting idea would be to study how these supports can have a protective effect on adolescent emotional and behavioral adjustment. Finally, the participants of the current study were only selected from three cities in China and did not sample for general population proportion, which may affect the representation of sample in the whole adolescents of China. Further studies should recruit participants from a wider geographical range and sampled for general population proportion. In spite of the above limitations, our study provided us with preliminary and innovative insights into the underlying mechanisms that influence the relationship between negative life events and antisocial behavior tendencies in adolescent.

## Conclusion

The adverse effect of negative life events on adolescent development has been well-documented. However, little is known about the internal influence mechanisms for the relationship between negative life events and antisocial behavior tendencies in adolescents. The present study again confirmed the promoting effect of negative life events on antisocial behavior tendencies in adolescents. Self-esteem was shown to be a mediator between negative life events and antisocial behavior tendencies. In addition, family support negatively moderated the indirect effect of negative life events on antisocial behavior tendencies *via* self-esteem. Negative life events had a greater indirect impact on antisocial behavior tendencies with poorer subjective family support. Subjective family support weakened the adverse effect of stressful life events on self-esteem, and consequently susceptibility to antisocial behavior in adolescents. These results displayed the important role of self-esteem and subjective family support in preventing antisocial behavior in adolescents. To minimize the adverse effect of negative life events on problem behavior development of adolescents, interventions are needed with the aim of raising self-esteem in adolescents, and parents need to be provided with support-skill training aimed at improving subjective family support of adolescents. This study came up with important implications for preventing antisocial behavior in adolescents. However, due to the cross-sectional design of the study, studies with a longitudinal design need to be conducted to further explore the causal relations between the variables studied.

## Data Availability Statement

The raw data supporting the conclusions of this article will be made available by the authors, without undue reservation.

## Ethics Statement

The studies involving human participants were reviewed and approved by The research ethics committee of Jinzhou Medical University (No: 2016-08). Written informed consent to participate in this study was provided by the participants’ legal guardian/next of kin.

## Author Contributions

FG made an outstanding contribution to the study design, data analysis and interpretation, and manuscript drafting and revision. YY participated in the study design, interpretation of the data, and manuscript revision as well. CY, YX, and HM assisted with organization of the research and data collection. HL helped with analyzing the data and drafting the manuscript. All authors contributed to the article and approved the submitted version.

### Conflict of Interest

The authors declare that the research was conducted in the absence of any commercial or financial relationships that could be construed as a potential conflict of interest.

## Abbreviations

ASLEC: Adolescent self-rating life events check list
CFA: Confirmatory factor analysis
CFI: Comparative fit index
TLI: Tucker-Lewis index
SRMR: The standardized root mean square residual
RMSEA: Root mean square error of approximation
CI: Confidence interval
SEM: Structural equation model
RSCA: Resilience scale for Chinese adolescent
MLR: Robust maximum likelihood estimator
LMS: Latent moderated structural equations
SD: Standard deviation.
